# The impact of a poverty reduction intervention on infant brain activity

**DOI:** 10.1073/pnas.2115649119

**Published:** 2022-01-24

**Authors:** Sonya V. Troller-Renfree, Molly A. Costanzo, Greg J. Duncan, Katherine Magnuson, Lisa A. Gennetian, Hirokazu Yoshikawa, Sarah Halpern-Meekin, Nathan A. Fox, Kimberly G. Noble

**Affiliations:** ^a^Department of Biobehavioral Sciences, Teachers College, Columbia University, New York, NY 10027;; ^b^Institute for Research on Poverty, University of Wisconsin–Madison, Madison, WI 53706;; ^c^School of Education, University of California, Irvine, CA 92697;; ^d^Sandra Rosenbaum School of Social Work, University of Wisconsin–Madison, Madison, WI 53706;; ^e^Sanford School of Public Policy, Duke University, Durham, NC 27708;; ^f^Department of Applied Psychology, New York University, New York, NY 10012;; ^g^School of Human Ecology and LaFollette School of Public Affairs, University of Wisconsin–Madison, Madison, WI 53706;; ^h^Department of Human Development and Quantitative Methodology, University of Maryland, College Park, MD 20742;; ^i^Department of Human Development, Teachers College, Columbia University, New York, NY 10027

**Keywords:** poverty, unconditional cash transfer, randomized control trial, infant brain activity, EEG

## Abstract

Early childhood poverty is a risk factor for lower school achievement, reduced earnings, and poorer health, and has been associated with differences in brain structure and function. Whether poverty causes differences in neurodevelopment, or is merely associated with factors that cause such differences, remains unclear. Here, we report estimates of the causal impact of a poverty reduction intervention on brain activity in the first year of life. We draw data from a subsample of the Baby’s First Years study, which recruited 1,000 diverse low-income mother–infant dyads. Shortly after giving birth, mothers were randomized to receive either a large or nominal monthly unconditional cash gift. Infant brain activity was assessed at approximately 1 y of age in the child’s home, using resting electroencephalography (EEG; *n* = 435). We hypothesized that infants in the high-cash gift group would have greater EEG power in the mid- to high-frequency bands and reduced power in a low-frequency band compared with infants in the low-cash gift group. Indeed, infants in the high-cash gift group showed more power in high-frequency bands. Effect sizes were similar in magnitude to many scalable education interventions, although the significance of estimates varied with the analytic specification. In sum, using a rigorous randomized design, we provide evidence that giving monthly unconditional cash transfers to mothers experiencing poverty in the first year of their children’s lives may change infant brain activity. Such changes reflect neuroplasticity and environmental adaptation and display a pattern that has been associated with the development of subsequent cognitive skills.

## Significance

This study demonstrates the causal impact of a poverty reduction intervention on early childhood brain activity. Data from the Baby’s First Years study, a randomized control trial, show that a predictable, monthly unconditional cash transfer given to low-income families may have a causal impact on infant brain activity. In the context of greater economic resources, children’s experiences changed, and their brain activity adapted to those experiences. The resultant brain activity patterns have been shown to be associated with the development of subsequent cognitive skills.

Early childhood poverty has long been associated with lower school achievement, educational attainment, and adult earnings (1–4). Moreover, from early childhood through adolescence, higher family income tends to be associated with higher scores on assessments of language, memory, self-regulation, and social-emotional processing (5–8). Furthermore, poverty has been correlated with the structural development and functional activity of brain regions that support these skills. For example, higher family income is associated with a larger surface area of the cerebral cortex, particularly in regions that support children’s language and executive functioning (9, 10). This association is strongest among the most economically disadvantaged families (9), suggesting that a given increase in family income may be linked with greater differences in brain structure among economically disadvantaged children compared with more advantaged peers (11).

Economic disadvantage has also been associated with differences in electrical brain activity, a key aspect of brain function that is measured by electroencephalography (EEG) (12–16). EEG measures brain activity along two primary dimensions: frequency and power. “Frequency” refers to oscillatory brain activity that occurs throughout the brain at different rates. Neuroscientists traditionally divide the continuous frequency spectrum into bands. Some of these bands represent lower-frequency (slower) oscillations (e.g., the theta-band), and some represent higher-frequency (faster) brain activity in the mid to high portions of the frequency spectrum (e.g., the alpha-, beta-, and gamma-bands). All individuals have brain activity across the frequency spectrum throughout the brain. “Power” refers to the amount of brain activity in a certain band measured across the scalp, broadly reflecting the electrical activity of the underlying brain. Power varies across frequency bands and between people. “Absolute power” refers to the amount of brain activity measured at a certain frequency (or within a certain frequency band). “Relative power” expresses absolute power as a fraction of power summed across all frequency bands.

Childhood EEG-based brain activity demonstrates a specific developmental pattern. As children mature from the neonatal period through middle childhood, they tend to show a decrease in brain power in the low-frequency portion of the frequency spectrum, as well an increase in brain power in the mid- to high-frequency portions of the frequency spectrum (17–20). Individual differences in this pattern, particularly in absolute power, have been associated with children’s cognitive and behavioral outcomes. For example, more absolute power in mid- to high- (i.e., alpha, beta, and gamma) frequency bands has been associated with higher language (21–24), cognitive (21, 25), and social-emotional (26) scores, whereas more absolute or relative low-frequency (i.e., theta) power has been associated with the development of behavioral, attention, or learning problems (27–29).

At birth, family income appears to be unrelated to brain activity, as measured by EEG (23). However, some studies find that family income quickly begins to predict differences in the neurodevelopmental patterns described above. Specifically, several studies with small sample sizes have suggested that within the first several years of life, children from lower-income families average more low-frequency (i.e., theta) EEG band power, and less mid- to high-frequency (i.e., alpha, beta, and gamma) band power compared with children from higher-income homes (13–15, 30). Similar patterns of more low-frequency band power and less mid- to high-frequency band power have also been found among children facing other forms of early adversity (31–33) and, in some of these studies, these differences appear to persist throughout childhood and early adolescence (13, 14, 34–36). Of course, these general patterns conceal considerable heterogeneity; not all children facing poverty or other forms of adversity will show evidence of these neurodevelopmental differences.

Neuroplasticity, or the concept that children’s brains adapt to their environmental contexts, is one path through which these differences are thought to emerge. That is, the structure and function of the developing brain adapt in response to different experiences. Brain activity may thus be one mechanism by which early adverse experiences shape subsequent child developmental outcomes.

Despite the correlational evidence linking income to early childhood cognitive development, it is unclear whether poverty causes developmental differences early in life (37). Support for a causal role comes from rigorous quasiexperimental studies that have linked increases in family income to higher school achievement and educational attainment, as well as to better physical and mental health (38). On the other hand, many other characteristics of individuals and their environments have been linked to these kinds of child outcomes (39). A careful experimental manipulation is needed to differentiate between these alternate interpretations.

The Baby’s First Years study (BFY; https://www.babysfirstyears.com) is the first randomized control trial of poverty reduction in early childhood, and was designed to address whether poverty reduction causes changes in children’s brain development (40). Based on prior economic research showing that relatively modest differences in early childhood family income are associated with better school achievement (41–43), BFY randomized 1,000 low-income mothers living in four geographically diverse United States metropolitan areas to receive either a large cash gift of $333 per month (termed the “high-cash gift group”) or a nominal cash gift of $20 per month (the “low-cash gift group”) for the first several years of their children’s lives. These cash gifts took the form of unconditional cash transfers provided on a debit card; participating mothers were told that the money could be used in any way they wished, with no restrictions. The $313/mo difference between the amount received by the high-cash and low-cash gift groups amounted to $3,756 per year. Here we report the differential impacts of these unconditional cash transfers on infant brain activity at 1 y of age. We preregistered our analytic plan and hypothesized that infants of mothers randomized to the high-cash gift group would show greater mid- to high-frequency (i.e., alpha, beta, gamma) power and decreased low-frequency (i.e., theta) power when compared with infants of mothers randomized to the low-cash gift group.

## Results

We recorded and analyzed the resting brain activity of 435 of the 1,000 infants whose mothers had been randomized to receive either a large monthly cash gift or a nominal monthly cash gift. (See *SI Appendix*, SI1 for a complete description of recruitment, retention, and EEG data collection procedures, including pandemic-related considerations with regard to in-person data collection.) Descriptive statistics for participant baseline characteristics are presented in Table 1. Mothers and infants were from racially and ethnically diverse backgrounds, with the majority of mothers identifying as Black or Hispanic. By design, all infants were healthy at birth (*SI Appendix*, SI1), and mothers reported average household incomes of just over $20,000 in the calendar year prior to the birth. On average, the cash gifts amounted to an approximate 20% boost in annual income for the mothers in the high-cash gift group.

**Table 1.** Characteristics of EEG sample

	Low-cash gift EEG sample		High-cash gift EEG sample		*P* value of group difference
		*n*		*n*	
Child is female	49.8	251	44.0	184	0.23
Child age at visit (mo)	12.93 (1.66)	251	12.60 (1.13)	184	0.02
Mother education (y)	11.9 (3.1)	248	12.1 (3.1)	183	0.60
Mother race/ethnicity					
White, non-Hispanic	11.6	251	6.0	184	0.05
Black, non-Hispanic	38.6	251	47.3	184	0.07
Multiple, non-Hispanic	5.6	251	2.7	184	0.15
Other or unknown	4.4	251	2.7	184	0.36
Hispanic	39.8	251	41.3	184	0.76
Household combined income at baseline (dollars)	$22,739 (20,875)	238	$20,213 (14,402)	168	0.18
Number of artifact-free EEG epochs	288.2 (183.7)	251	284.3 (189.2)	184	0.83

Data are presented as mean (SD) or %. Child age and number of epochs were measured at the time of the age 1 visit. All other characteristics were measured at baseline prior to random assignment. Household income measures are as reported by mother at time of baseline. This includes two outlier values in the low-cash gift group (>3 SD above the mean), which results in the large SD for the low-cash gift group for the household income measure. Reported *P* values of mean differences are unadjusted. For site-adjusted *P* values and a joint test of orthogonality for baseline measures, see *SI Appendix*, Table SI1.1.

In order to compare age-1 brain activity of infants in the high-cash and low-cash gift groups, intent-to-treat (ITT) analyses were conducted on absolute and relative EEG power in four power bands: theta, alpha, beta, and gamma. (See *SI Appendix*, SI2 for information on EEG processing, *SI Appendix*, SI3 for a discussion of absolute vs. relative power, and *SI Appendix*, SI4 for information on preregistration and hypotheses.) Table 2 shows these ITT estimates before and after adjustments for baseline covariates and multiple hypothesis testing. The effect size column standardizes each adjusted coefficient by dividing it by the SD of the given outcome measure within the low-cash gift group in the *n* = 435 EEG sample. The study was originally designed to have the statistical power to detect an effect size of 0.21 SD for any single hypothesis (*SI Appendix*, SI4). Despite the relatively small departures in baseline balance between the high-cash and low-cash groups shown in Table 1, we note that some of the ITT estimates change when covariates are added to the models.

**Table 2.** Cash-gift treatment effects on EEG power

	Low-cash gift group mean (SD)	High-cash gift group mean (SD)	OLS with site fixed effects (SE)	OLS with site fixed effects and covariates (SE)	Effect size (including covariates)	*P* value (no adjustments)	Westfall–Young adjusted *P* value	*n*
Absolute alpha	7.441 (4.213)	7.667 (3.896)	0.294 (0.381)	0.720 (0.396)	0.17	0.07	0.12	435
Absolute beta	1.874 (1.592)	2.167 (2.281)	0.307 (0.187)	0.414 (0.176)	0.26	0.02	0.07	435
Absolute gamma	0.986 (0.947)	1.137 (1.202)	0.155 (0.103)	0.221 (0.109)	0.23	0.04	0.12	435
Absolute theta	40.268 (23.317)	38.887 (16.578)	−0.961 (1.860)	0.396 (1.869)	0.02	0.83	0.84	435
Relative alpha	0.148 (0.040)	0.152 (0.045)	0.004 (0.004)	0.006 (0.005)	0.16	0.17	0.31	435
Relative beta	0.038 (0.027)	0.042 (0.036)	0.004 (0.003)	0.005 (0.003)	0.19	0.09	0.19	435
Relative gamma	0.020 (0.018)	0.022 (0.021)	0.002 (0.002)	0.003 (0.002)	0.16	0.18	0.31	435
Relative theta	0.794 (0.070)	0.784 (0.083)	−0.010 (0.007)	−0.014 (0.008)	−0.21	0.07	0.17	435

OLS, ordinary least squares. Effect size (column 5) was computed by dividing the covariate-adjusted treatment effect (column 4) by the SD of the EEG sample low-cash group. Unadjusted *P* values (column 6) and preregistered Westfall–Young adjusted *P* values (column 7), which adjust for multiple hypothesis testing, are both reported. For the Westfall–Young adjustment, the four frequency bands (theta, alpha, beta, gamma) for absolute power are placed into one family and the four frequency bands (theta, alpha, beta, gamma) for relative power were placed into a second family. These *P* values are associated with the treatment coefficient and effect size in a regression with site-level fixed effects and covariates. Covariate-adjusted models include the following maternal self-report covariates from the BFY baseline survey conducted at the time of enrollment: mother’s age, completed maternal schooling, household income, net worth, general maternal health, maternal mental health, maternal race and ethnicity, marital status, number of adults in the household, number of other children born to the mother, maternal smoking during pregnancy, maternal alcohol consumption during pregnancy, father living with the mother, child’s sex, child’s birth weight, child’s gestational age at birth. Models also control for child’s age at interview (in months), and the total number of usable epochs. Missing data for covariates impute the mean value from the EEG analytic sample. Relative power calculated at the child-level. Robust SEs are given in parentheses for OLS models (columns 5 and 6). SDs provide in parentheses in columns 1 and 2.

In the case of absolute power, the high-cash gift group showed higher power in the three mid- to high-frequency bands (alpha, beta, and gamma) but not in the low-frequency theta-band (top rows of Table 2). When ranked by effect sizes, group differences in EEG power in the beta-band were largest (effect size = 0.26, beta = 0.414, *P* = 0.02, for the model with covariates and site fixed effects), followed by the gamma-band (effect size = 0.23, beta = 0.221, *P* = 0.04). Both *P* levels were below the 0.05 threshold when treated as independent measures, but not after Westfall–Young (44) multiple-testing adjustments. Group power differences in the alpha-band (effect size = 0.17, beta = 0.720, *P* = 0.07) were smaller and at the margins of statistical significance. Small and statistically nonsignificant differences in absolute power were found in the theta-band (effect size = 0.02, beta = 0.396, *P* = 0.83). (See *SI Appendix*, SI5 for a similar pattern in weighted analyses that adjust for demographic differences between the *n* = 435 EEG sample and the *n* = 931 full sample of BFY mother/infant dyads interviewed at age 1.)

Differences in relative power were qualitatively similar but uniformly smaller than those observed for absolute power, with the high-cash gift group showing greater mid- to high-frequency relative power in the alpha-, beta-, and gamma-bands. These differences did not reach conventional levels of statistical significance (bottom rows of Table 2; for a more complete discussion of absolute and relative power, see *SI Appendix*, SI3). In contrast, relative theta-power was greater in the low-cash gift group with an effect size of 0.21, with the difference at the margins of statistical significance (*SI Appendix*, SI4).

**Fig. 1.**
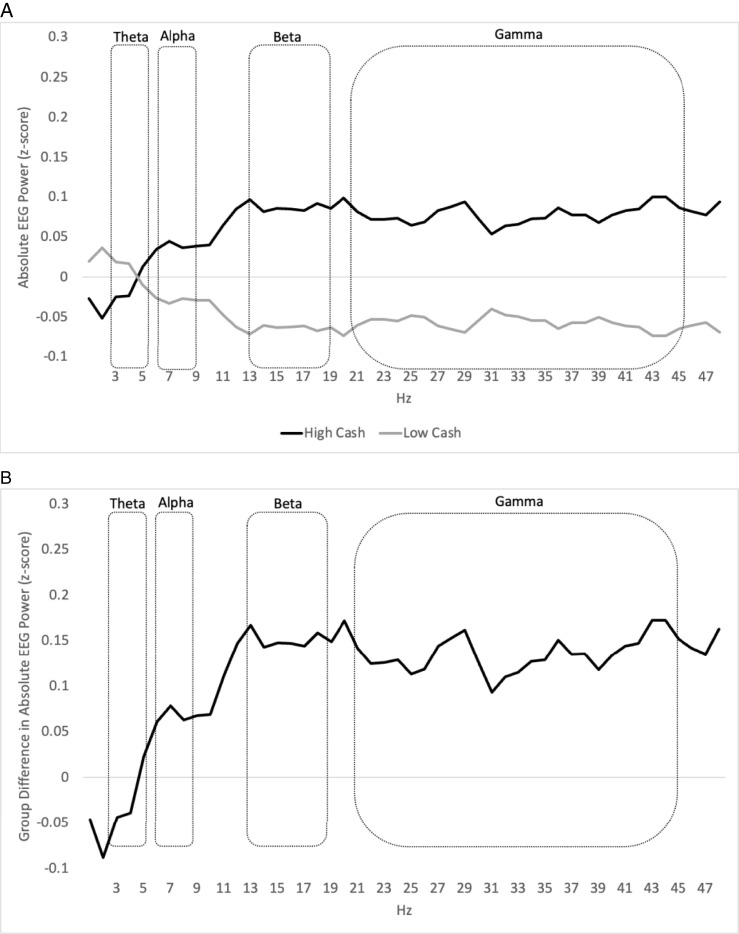
(*A*) Standardized mean absolute EEG power is presented separately for the high-cash and low-cash gift groups. The high-cash gift group’s means are depicted with a solid black line and the low-cash gift group’s means are depicted with a solid gray line. The power spectrum is displayed continuously with single-hertz bins on the *x* axis, standardized absolute power on the *y* axis, and with the boundaries of the preregistered theta-, alpha-, beta-, and gamma-frequency bands delineated, demonstrating that the pattern of results is consistent across the spectra and that a small number of single-hertz bins did not unduly impact the results shown in Table 2. Because power values were standardized (*z*-scored) using the mean and SD of the entire *n* = 435 sample, the two lines are mirror images of one another. This graph is intended for illustrative purposes only and does not include adjustment for covariates; statistical testing was conducted on aggregations of single-hertz bin values within a given frequency band (e.g., theta). (*B*) The difference between standardized EEG absolute power (*z*-scores) in the high-cash vs. low-cash gift groups is depicted with a solid black line. The power spectrum is displayed continuously with single-hertz bins on the *x* axis, group differences in standardized on the *y* axis, and with the boundaries of the preregistered theta-, alpha-, beta-, and gamma-frequency bands delineated, demonstrating that the pattern of results is consistent across the spectra and that a small number of single-hertz bins did not unduly impact the results shown in Table 2. This graph is intended for illustrative purposes only and does not include adjustment for covariates; statistical testing was conducted on aggregations of single-hertz bin values within a given frequency band (e.g., theta) and is shown in Table 2.

**Fig. 2.**
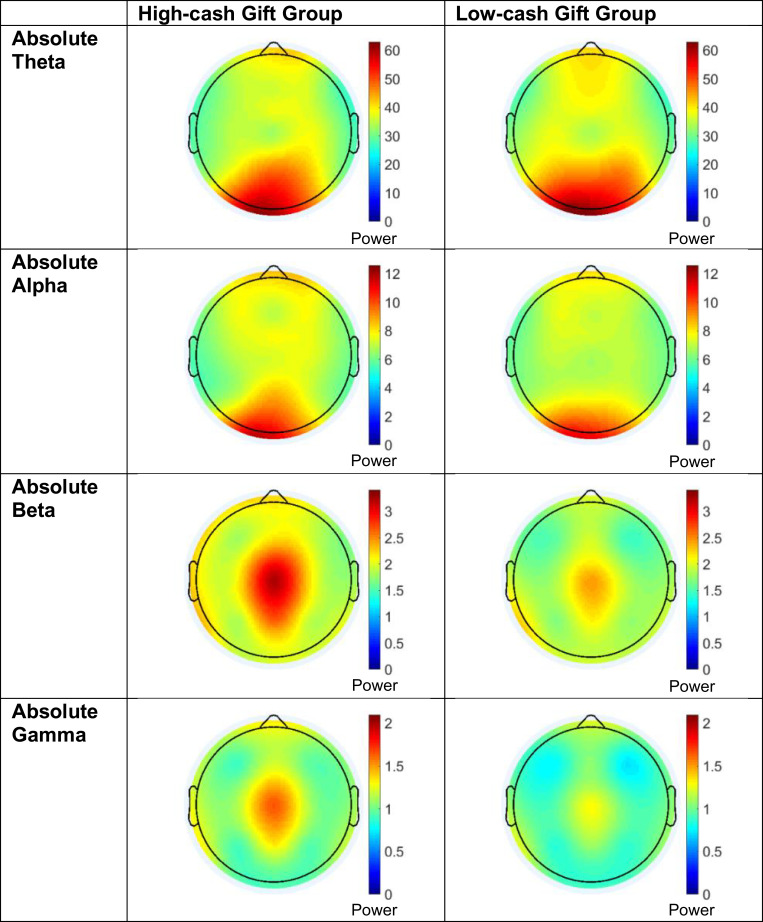
Topographic heat maps show the distribution of absolute theta-, alpha-, beta-, and gamma-power across the scalp for the high-cash gift group (*Left*) and low-cash gift group (*Right*). Warmer colors represent more power in each respective frequency band. Heat maps also illustrate the absence of any major artifact (e.g., remaining eye blinks). Regional differences are explored in *SI Appendix*, SI6. Additionally, because the EEG data are referenced to an average of the T7 and T8 electrodes, the temporal data are estimated from the surrounding electrodes for visualization purposes only.

Figs. 1 and 2 display the differences between absolute power brain activity in the high-cash gift group and the low-cash gift group across the frequency spectrum and across the scalp. Specifically, Fig. 1*A* displays *z*-scores of absolute EEG power across the full power spectrum separately for infants in the high-cash and low-cash gift groups, while Fig. 1*B* shows the corresponding group differences in *z*-scores across the power spectrum. Fig. 2 shows a topographic heat map of the distribution of EEG absolute power across the scalp within each of the four power bands, separately for the high-cash and low-cash gift groups.

Power data plotted in Fig. 1 are standardized (*z*-scored) based on the full EEG sample within each of the 48 single-hertz bins, with the boundaries of the theta-, alpha-, beta-, and gamma-frequency bands delineated. Given the standardization, the vertical distance between the two lines in Fig. 1*A* reflects standardized differences between infants in the high-cash and low-cash gift groups. These differences in *z*-scores are shown in Fig. 1*B*. Absolute power in the high-cash gift group is estimated to exceed absolute power in the low-cash gift group in all mid- to high-frequency single-hertz bins above 6 Hz: that is, including the entirety of the alpha-, beta-, and gamma-portions of the frequency spectrum.

Fig. 2 reinforces these differences by displaying the distribution of power across the scalp for both groups in each frequency band. Warmer colors represent more power in each respective frequency band, illustrating that the high-cash gift group appears to show more beta- and gamma-power relative to the low-cash gift group. Exploratory post hoc regional analyses are broadly consistent with the group differences illustrated in Fig. 2. Both before and after Westfall–Young adjustment, the high-cash gift group shows more frontal absolute beta-power (effect size = 0.32, beta = 0.46, *P*_unadjusted_ = 0.01, *P*_adjusted_ = 0.02); more central absolute beta-power (effect size = 0.28, beta = 0.59, *P*_unadjusted_ = 0.02, *P*_adjusted_ = 0.05); and more frontal absolute gamma-power (effect size = 0.26, beta = 0.238, *P*_unadjusted_ = 0.02, *P*_adjusted_ = 0.04) (*SI Appendix*, SI6).

Given our hypotheses of positive differences across all mid- to high-frequency portions of the power spectrum, we aggregated power across all three of our preregistered mid- to high-frequency power bands. Such a summary index approach is a commonly used data-reduction technique in the social sciences (45, 46), and serves as a post hoc complement to our preregistered Westfall–Young multiple comparison adjustment. While this approach ignores the biological and functional significance of the EEG bands, it has the benefit of enabling us to statistically estimate ITT differences for a single aggregated mid- to high-frequency index score (*SI Appendix*, SI7). Consistent with our band-based results, we find that the infants in the high-cash gift group had more mid- to high-frequency band absolute power than infants in the low-cash gift group (effect size = 0.25, beta = 13.35, *P* = 0.02) (*SI Appendix*, Table SI7.1). Thus, the direction and approximate size of intervention effects on mid- to high-frequency absolute power are similar when power is analyzed in preregistered bands, disaggregated into single-hertz bins, examined within regions or aggregated across bands.

## Discussion

While family income has been found to be associated with developmental differences in children’s brain structure and function, there is considerable debate as to whether growing up in poverty causes differences in early brain development, or whether poverty is merely correlated with other factors that are the true cause of early differences (37). Here, using a randomized control trial design, we offer evidence on this correlation vs. causation debate by showing that an intervention designed to reduce poverty appeared to cause changes in children’s brain functioning in ways that have been linked to subsequent higher cognitive skills.

Specifically, infants whose mothers were randomized at the time of their birth to receive a large monthly unconditional cash transfer showed greater mid- to high-frequency absolute EEG power in the alpha-, beta-, and gamma-bands (effect sizes = 0.17 to 0.26), compared with infants whose mothers were randomized to receive a nominal monthly unconditional cash transfer. In contrast, our findings do not provide consistent support for the hypothesis that the high-cash gift group would show less low-frequency power in the theta-band.

Impact estimates for each of the three mid- to high-frequency power bands were uniformly positive, with the high-cash gift group displaying higher power values than the low-cash gift group (Fig. 1 and Table 2). In the case of absolute power for the beta- and gamma-bands, the magnitudes of effect sizes were consistent with those that the study was designed to be able to detect for independent hypotheses (*SI Appendix*, SI4). Notably, however, estimates of the effect of the cash gift in these two highest-frequency bands were statistically significant before, but not after, adjustments for multiple comparisons.

To investigate the robustness of these findings, we consider three additional forms of evidence. First, when disaggregating the mid- to high-frequency (alpha, beta, and gamma) portion of the spectrum into single-hertz bins, we found that infants in the high-cash gift group display higher power than infants in the low-cash gift group, across the entire frequency spectrum from 6 to 49 Hz (Fig. 1). Second, the neural regions driving these impacts (Fig. 2 and *SI Appendix*, SI6) are broadly consistent with those reported in previous correlational work linking income to brain activity (13–15, 24, 35) and linking brain activity to language (21, 22) and cognitive outcomes (23, 25). Some of these fronto-central regional effects in the beta- and gamma-bands remain significant after adjusting for multiple comparisons (*SI Appendix*, Table SI6.1). Third, similar group differences were found for a post hoc composite index of mid- to high-frequency power, with infants in the high-cash gift group having significantly higher values on this index score than infants in the low-cash gift group (*SI Appendix*, Table S17.1). But while most of our evidence points to a plausible causal impact of the cash gifts, not all evidence presented here survives stringent multiple comparison correction, precluding full confidence in being able to reject the null hypotheses. Caution and further replication are therefore clearly warranted.

On balance, though, we judge that the weight of the evidence supports the conclusion that monthly unconditional cash transfers given to the mothers in our study affected brain activity in their infants. This is notable because the patterns of neural activity we observe in the high-cash gift group have been correlated with higher language (21–24), cognitive (21, 25), and social-emotional (26) scores later in childhood and adolescence. Moreover, the observed effects in the alpha-, beta-, and gamma-bands are similar in magnitude to those reported in other large-scale environmental interventions. For example, a meta-analysis of 747 randomized control trials of educational interventions targeting standardized achievement outcomes found an average effect size of 0.16 SDs (47).

Children’s brain development reflects an adaptation to their lived experiences (48, 49). Importantly, different brain activity patterns are likely to be adaptive in different contexts, and a typically developing brain will adapt to the environment it experiences (50). In some cases, such malleability may confer obvious benefits, whereas in other cases, it may lead to the development of adaptive but costly strategies for optimizing biological fitness under scarce conditions (51). In the latter case, adaptation does not necessarily represent dysfunction or dysregulation, but rather, an expected and appropriate response to the environment (52).

The present study provides evidence of neuroplasticity of the infant brain on a relatively brief time scale, following 1 y of an intervention designed to increase family economic resources. Because of the randomized design, any group differences in brain activity found here reflect neural adaptation to the associated environmental change. That is, in the context of greater economic resources, children’s experiences changed, and their brain activity adapted to those experiences. However, we do not yet know which experiences were involved in generating these impacts. Future work will examine potential mechanisms affected by the cash gifts, including household expenditures, maternal labor market participation, maternal parenting behaviors, and family stress, noting that pathways may operate in different ways across different children and families.

Several limitations should be noted when interpreting these results. First, the extent to which individual differences in infant brain activity are stable over time is not yet known (53). Second, because of the pandemic, EEG data could not be collected on the full *n* = 1,000 study sample. Although recruitment had been designed to provide comparable samples of participants across the recruitment year, the pandemic truncated our in-person data-collection effort, reducing the sample size considerably and decreasing the precision of our estimates. The extent to which the results presented here would have generalized to the full study sample is unknown (*SI Appendix*, SI5 and SI8). Third, we do not know whether the neurodevelopmental effects of this poverty reduction intervention will translate into differences in direct assessments of children’s skills and behavior. While associations between infant brain activity and subsequent cognitive, linguistic, and social-emotional functioning have been observed in other samples (22, 23, 25, 26), some studies do not find that infant brain activity predicts subsequent skills (22, 26). The BFY study will continue to follow these children through at least the first 4 y of life, to determine whether treatment impacts on brain activity persist and extend to direct measures of children’s cognitive and behavioral outcomes.

Despite the limitations in statistical power, the pattern of impacts, which resulted from a rigorous random assignment study design, were consistent with hypotheses, were similar in magnitude to effects on cognitive outcomes from other scalable interventions, and were largely robust to various tests (*SI Appendix*, SI4–SI9), leads us to conclude that these findings are important and unlikely to be spurious.

The present results suggest that providing monthly unconditional cash support to families living in poverty may impact early childhood brain activity, highlighting the importance of centering children’s development and well-being at the forefront of policy considerations. However, while it might be tempting to draw policy conclusions, we caution that the present findings pertain only to the first 12 mo of a multiyear unconditional cash transfer intervention. Recent legislation and policy proposals provide income supplements to low-income families in the form of Child Tax Credit payments with higher payments in early childhood, but none would limit assistance to the first year of life (54). For our part, we do not suggest that a 12-mo intervention alone would be likely to have lasting effects, nor that cash transfer policies obviate the need for direct service interventions, such as well-child pediatric visits, home visitation, or high-quality early childhood education. Nonetheless, by targeting families during children’s earliest years, BFY has found important evidence of the effects of increased income during a time when children’s brains are particularly sensitive to experience. Traditionally, debates over income transfer policies directed at low-income families in the United States have centered on maternal labor supply rather than child well-being. Our findings underscore the importance of shifting the conversation to focus more attention on whether or how income transfer policies promote children’s development.

## Materials and Methods

### Participants.

One thousand mother/infant dyads were enrolled in BFY over a 13-mo period beginning in May 2018. Mothers were recruited in hospital postpartum wards in four United States metropolitan areas: New York City, the greater New Orleans metropolitan area, the greater Omaha metropolitan area, and the Twin Cities (Minneapolis and St. Paul) metropolitan area. Shortly after giving birth, 40% of the mothers were randomly chosen to receive a large monthly cash gift of $333 per month (high-cash gift group) and the remaining 60% received a nominal monthly cash gift of $20 per month (low-cash gift group) for the first several years of their children’s lives. Random assignment was a continuous process over the enrollment period. At the time of enrollment, the mothers were told that the monthly cash gifts would continue for 40 mo, and that the study team would follow up with them annually for the next 3 y to assess child development and family life. Subsequently, the cash gifts were extended for an additional 12 mo, through child age 52 mo, and planned follow-up was extended through at least a 4-y period. Prior to launching the study, we secured approvals from state or local officials to ensure that participants would not lose eligibility for most public benefits due to the cash gift. The Institutional Review Boards of Teachers College, Columbia University; the University of California, Irvine; and the New York State Psychiatric Institute approved this study. Informed consent was collected by trained interviewers via an electronic consent form that was read to participants either in person or over the phone (consent collection method was consistent with the method of administration for the maternal survey). For more information concerning eligibility criteria, study design, and baseline data see https://www.babysfirstyears.com, Noble et al. (40), and the Interuniversity Consortium for Political and Social Research (ICPSR) data repository (55).

The present study centers on those infants from whom data were collected during the 1-y visit (mean = 12.92 mo, SD = 1.89). Initially, these 1-y visits were conducted in families’ homes. However, because of the COVID-19 pandemic and concerns for participant and interviewer safety, in-person data collection was halted on March 14, 2020, a point at which roughly two-thirds of the recruited infants had reached 12 mo of age. At that time, the survey data collection mode switched from in-person (*n* = 605) to phone (*n* = 326). All age-1 measures requiring in-person assessment were suspended at that point, including measures of infant brain activity. In total, 931 mothers eventually completed the age-1 survey (93% completion rate; complete survey information available at https://www.babysfirstyears.com) but only 605 were interviewed in the home, making their infants potentially eligible for EEG-based data collection.

Given that the focus of the present study is on infant brain activity, our primary analyses are limited to the 435 families who completed in-person EEG data collection with usable data prior to the onset of the pandemic (mean_age_= 12.79 mo, SD= 1.47) (see *SI Appendix*, SI1 for CONSORT diagram; *SI Appendix*, SI5 and SI8 for more information on the generalizability of findings in the prepandemic sample to the full sample; and *SI Appendix*, SI8 and SI10 for information about maternal report of infant developmental milestones, which are available for the full sample).

### EEG Data Collection.

To assess brain activity, EEG data were collected using a mobile system in the home. The utility, feasibility, and cultural appropriateness of mobile EEG were evaluated prior to the commencement of data collection through a series of pilot visits and focus groups [see Troller-Renfree et al. (56) for full details of piloting and interviewer training]. Following this piloting process, a team of interviewers was trained to collect in-home EEG.

EEG was recorded using a 20-channel Neuroelectrics cap with an Enobio 20 amplifier (Neuroelectrics). The sampling rate was 500 Hz and data were referenced online to a DRL/CMS reference configuration placed on or near the mastoid bone. During the recording, infants sat on their caregivers’ laps while watching infant-friendly wordless videos or observing bubbles or infant toys. Recordings lasted a maximum of 7 min with a goal of recording at least 5 min of artifact-free data. Data were analyzed off-line by data processors who were blind to participant group (See *SI Appendix*, SI2, SI3, and SI9 for information on EEG data processing and analysis).

Of the 605 participants who completed age-1 visits before the onset of the pandemic, 577 mothers consented to EEG data collection (95.4% consent rate). A total of 142 infants of these consenting mothers did not contribute a usable EEG recording, for reasons including infant fussiness (*n* = 62), excessive artifact during recording (*n* = 52), technical problems (*n* = 16), poor cap fit (*n* = 9), and interviewer error (*n* = 3). Ultimately, usable data were obtained from 435 infants for analysis (75.4% of participants who consented to EEG collection). The heat maps in Fig. 2 illustrate the absence of any major artifact (e.g., remaining eye blinks).

### Preregistration and Statistical Analysis.

In keeping with its randomized control trial study design, BFY preregistered data collection and analysis plans (ClinicalTrials.gov Identifier: NCT03593356; for more information about preregistered analyses and hypotheses, see *SI Appendix*, SI4). Consistent with our preregistration and in light of the nearly universal take-up of our cash gifts in both high-cash and low-cash gift group families, ITT differences were estimated using a simple regression framework. All models were estimated using robust SEs (57) and estimated ITT differences without, and then with, baseline demographic child and family characteristics to improve the precision of our estimates.

## Supplementary Material

Supplementary File

## Data Availability

Anonymized data have been deposited in ICPSR, https://www.icpsr.umich.edu/web/DSDR/studies/37871/versions/V2 (55) and https://www.openicpsr.org/openicpsr/project/159422/ (58).

## Change History

January 25, 2022: The article text has been updated.
