# Supplementary material for: The impact of a poverty reduction intervention on infant brain activity
Source: Proc Natl Acad Sci U S A. 2022 Jan 24;119(5):e2115649119. doi: 10.1073/pnas.2115649119 (PMC8812534; doi:10.1073/pnas.2115649119)
Supplement: Supplementary File [file pnas.2115649119.sapp.pdf]

Supplemental Information

Here we provide supplemental information to accompany the main manuscript. In SI1 we explain participant recruitment and examine baseline balance. SI2 provides details on our EEG data processing and analysis. In SI3 we discuss similarities and differences between absolute and relative power measures. In SI4 we discuss our preregistration procedures and hypotheses. In SI5 we conduct weighted analyses to assess the robustness of our results to (1) differences in the composition of the sample at baseline across treatment groups, and (2) differences in the composition of the EEG sample compared with the full study sample. In SI6 we present ITT impacts on regional EEG power. In SI7 we present a robustness check, in the form of a social science-style summary index, summing across the mid-to-high-frequency pre-registered bands. In SI8 we present ITT impacts on a behavioral complement to the measures of brain activity, the Ages and Stages Questionnaire-3 (ASQ-3) Communication Subscale. This subscale of the ASQ-3 screens for language delay, by asking mothers to report on the child's language milestones. We additionally examine differences in ASQ-3 scores between the EEG subsample and full analytic sample to assess the robustness of our results. In SI9 we provide a final robustness check on our EEG results by showing ITT impacts on the log-transformed EEG power spectrum. In SI10 we investigate the relations between EEG power and infant language milestones.

SI1. Participant recruitment and age-1 follow-up

Baby's First Years (BFY) was designed to estimate the causal impact of a poverty reduction intervention on children's early development (see Noble et al., 2021 (1) for complete details on study design). Between May 2018 and June 2019, 1,000 mother-infant-dyads were recruited to participate in the BFY study. BFY sample recruitment was restricted to mothers of newborns whose self-reported income in the prior calendar year was below the federal poverty line. Additional study inclusion criteria were: (1) mother was of legal age for informed consent (age 18 or older in NY, MN and LA; 19 or older in NE); (2) infant was admitted to the newborn nursery, and not the neonatal intensive care unit; (3) mother was residing in the state of recruitment (needed to ensure the cash gift would not be counted in determining eligibility for that

state's public antipoverty benefits); (4) mother reported not being "highly likely" to move to a different state or country within 12 months; (5) infant was discharged in the custody of the mother; and (6) mother spoke either English or Spanish. Using a joint test, mother-infant dyads assigned to the high-cash and low-cash gift groups were shown to be comparable across a large set of demographic characteristics gathered at baseline just prior to random assignment (Noble et al., 2021 (1); CONSORT diagram Figure SI1.1). Mothers were randomly assigned within each of the four sites to either the high-cash gift condition of $333/month (40% of the sample) or low-cash gift condition of $20/month (60% of the sample). The cash gifts were disbursed on debit cards branded 4MyBaby, which were activated in the hospital at the time of recruitment, while the mother was in the postpartum ward. Mothers received monthly text messages to alert them each month when the cards were reloaded.

Figure SI1.1. Consort diagram.

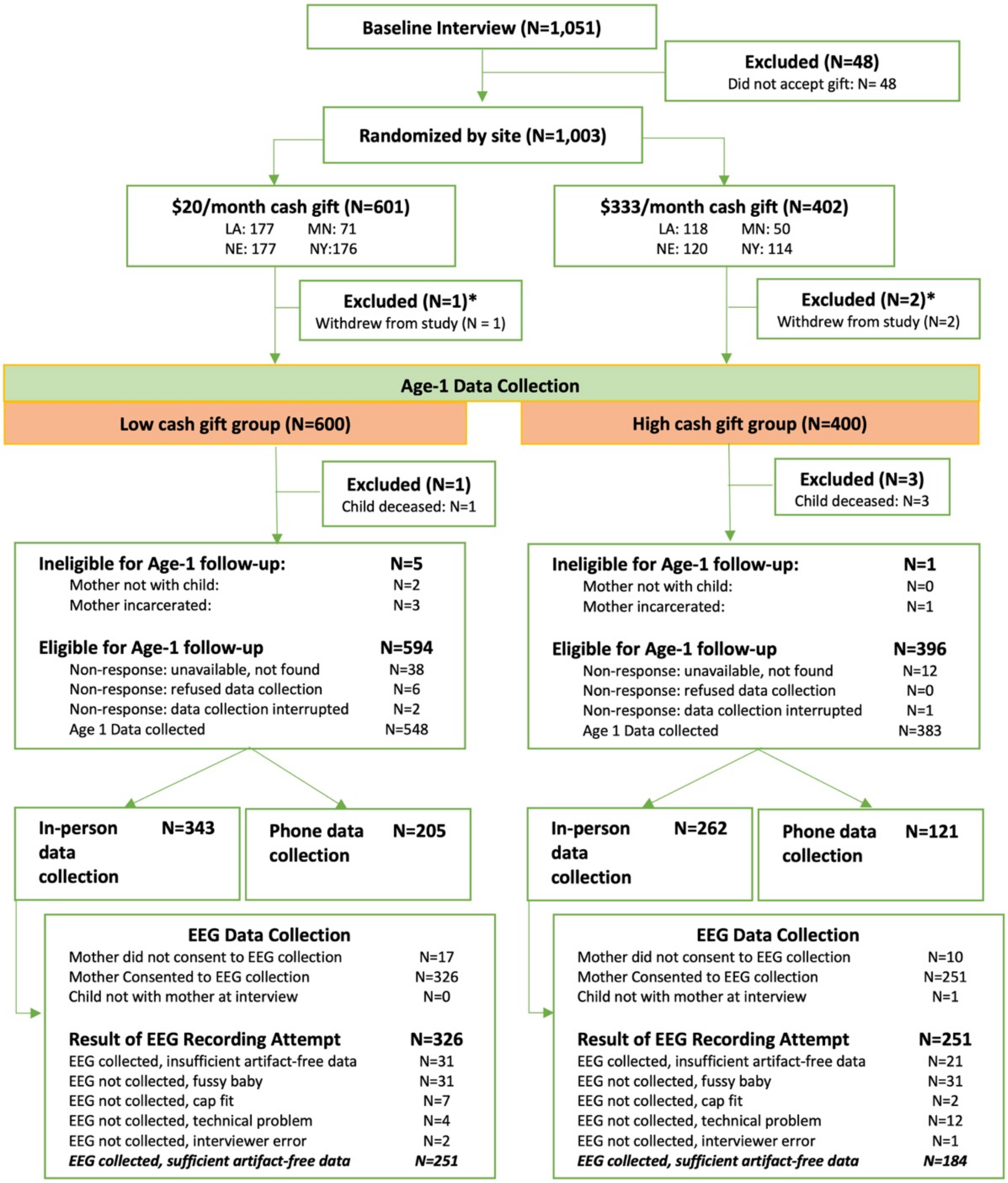

*Participants withdrew from study prior to spending any money on card and only a few days after randomization. Thus, they were not considered as the **target sample** for future waves of data collection.

Between July 2019 and July 2020, we attempted to contact as many of the 1,000 study participants as possible and interview them close as possible to their children's first birthdays. We completed interviews with 931 participants (see below). However, as explained in the main text,

in-home interviews were completed with only 605 families. Age-1 data collection rates are also summarized in the CONSORT Figure SI1.1.

From the Age 1 in-home visits, usable EEG data were obtained from 435 of the 605 infants who were tested in person. We assessed whether the baseline characteristics of the 435 differed from the 170 infants tested from whom usable EEG data were not obtained (Table SI1.1). A joint test of differences across all of the baseline measures fell well short of conventional levels of statistical significance (Joint Test: $Chi^2(25) = 30.25$, p-value = 0.22, n = 605), suggesting the two groups were broadly similar. For comparisons of individual characteristics, see Table SI1.1.

At the point in-person data collection was halted, we had a somewhat higher response rate among the high-cash gift group (66%) than the low-cash gift group (57%). To investigate the possible implications of this for our analysis sample, we examined balance across baseline characteristics between the high-cash and low-cash gift groups for the subset of children who contributed usable EEG data (SI Table 1.2). Here again, few differences were apparent and a joint test across all of the baseline measures showed a *p* value of .09 (joint test: $Chi^2(26) = 36.10$, p-value = 0.09, n = 435). For comparisons of individual characteristics, see Table SI1.2.

Table SI1.1 Balance on baseline characteristics comparing children who had usable EEG data and children who had in-person data collection but contributed no usable EEG data.

	Non-EEG In-Person Sample		EEG Sample		Std Mean Difference		
	Mean (sd)	N	Mean (sd)	N	Hedges' g	Cox's Index	p-value
Child is female	0.500	170	0.474	435		-0.06	0.54
Child weight at birth (pounds)	7.1 (1.0)	170	7.1 (1.0)	434	0.06		0.49
Child gestational age (weeks)	39.0 (1.1)	170	39.1 (1.4)	432	0.07		0.42
Mother age at birth (years)	26.7 (5.0)	170	27.2 (6.1)	435	0.08		0.26
Mother education (years)	11.8 (2.7)	169	12.0 (3.1)	431	0.06		0.52
Mother race/ethnicity: white, non-Hispanic	0.106	170	0.092	435		-0.10	0.51
Mother race/ethnicity: Black, non-Hispanic	0.447	170	0.423	435		-0.06	0.21
Mother race/ethnicity: multiple, non-	0.041	170	0.044	435		0.05	0.92

Hispanic							
Mother race/ethnicity: other or unknown	0.041	170	0.037	435		-0.07	0.61
Mother race/ethnicity: Hispanic	0.365	170	0.405	435		0.10	0.03
Mother marital status: never married	0.500	170	0.467	435		-0.08	0.32
Mother marital status: single, living with partner	0.253	170	0.241	435		-0.04	0.73
Mother marital status: married	0.194	170	0.207	435		0.05	0.58
Mother marital status: divorced/separated	0.018	170	0.041	435		0.51	0.06
Mother marital status: other or unknown	0.035	170	0.044	435		0.14	0.63
Mother health is good or better	0.871	170	0.910	435		0.24	0.18
Mother depression (CESD)	0.7<br>(0.4)	170	0.7<br>(0.4)	435	-0.03		0.54
Cigarettes per week during pregnancy	4.7<br>(16.6)	170	4.3<br>(18.6)	432	-0.02		0.68
Alcohol drinks per week during pregnancy	0.0<br>(0.0)	170	0.1<br>(0.6)	433	0.11		0.06
Number of children born to mother	2.6<br>(1.4)	170	2.4<br>(1.4)	435	-0.11		0.19
Number of adults in household	2.0<br>(0.9)	170	2.1<br>(1.0)	435	0.07		0.41
Biological father lives in household	0.429	170	0.340	435		-0.23	0.04
Household combined income	$21,189<br>(17,663)	160	$21,694<br>(18,496)	406	0.03		0.89
Household income unknown	0.059	170	0.067	435		0.08	0.69
Household net worth	-$3,386<br>(12,763)	154	-$1,495<br>(32,496)	386	0.07		0.31
Household net worth unknown	0.094	170	0.113	435		0.12	0.39

Joint Test: $Chi^2(25)$= 30.25, p-value= 0.22, n=605. (includes all observations. Standard joint test estimate drops 9 observations due to collinearity in a small number of observations with values for child's weight unknown, gestational age unknown, and mother's cigarette and alcohol use unknown)

Notes: "sd" = standard deviation. P-values were derived from a series of OLS bivariate regressions in which each respective baseline characteristic was regressed on the treatment status indicator using robust standard errors and site-level fixed effects. The bivariate regressions were also run without site-level fixed effects, and the p-values differed on average by 0.050 but result in no difference in substantive understanding or statistical significance interpretation. The p-values without fixed effects do not appear in the table. The joint test of orthogonality was conducted using a probit model with robust standard errors and site-level fixed effects. Standardized mean differences were calculated using Hedge's g for continuous variables and Cox's Index for dichotomous variables. If there were more than 10 missing cases for a covariate, missing data dummies were included in the table and the joint test. If there were fewer than 10 cases missing, missing data dummies were not included in the table but were included in the joint test; additionally, the joint test imputes mean values for missing variables. Chi-square tests of independence were conducted for the two categorical variables: mother race/ethnicity and mother marital status. For both tests, p>0.05. All respondents with missing data for baseline variables for child's weight, gestational age, mother's cigarette use, and mother's alcohol use were in the EEG sample, perfectly predicting. We present the results of the joint test that include these observations and exclude these variables for the full sample (which would be dropped in the standard test due to collinearity). If we instead remove these observations from the sample for the joint-test, the sample for the joint-test is slightly reduced, and the estimates are as follows: Joint Test: $Chi^2(25)$ = 30.74, p-value = 0.20, n = 596.

Table SI1.2 Balance on baseline characteristics between the low-cash and high-cash gift groups for children who had usable EEG data.

	Low-Cash EEG Sample		High-Cash EEG Sample		Std Mean Difference		
	Mean (sd)	N	Mean (sd)	N	Hedges' g	Cox's Index	p-value
Child is female	0.498	251	0.440	184		-0.14	0.23
Child weight at birth (pounds)	7.1 (1.0)	250	7.2 (1.0)	184	0.14		0.12
Child gestational age (weeks)	39.1 (1.3)	248	39.0 (1.4)	184	-0.02		0.98
Mother age at birth (years)	26.8 (6.1)	251	27.7 (6.2)	184	0.13		0.11
Mother education (years)	11.9 (3.1)	248	12.1 (3.1)	183	0.05		0.58
Mother race/ethnicity: white, non-Hispanic	0.116	251	0.060	184		-0.44	0.02
Mother race/ethnicity: Black, non-Hispanic	0.386	251	0.473	184		0.22	0.12
Mother race/ethnicity: multiple, non-Hispanic	0.056	251	0.027	184		-0.46	0.10
Mother race/ethnicity: other or unknown	0.044	251	0.027	184		-0.31	0.30
Mother race/ethnicity: Hispanic	0.398	251	0.413	184		0.04	0.25
Mother marital status: never married	0.426	251	0.522	184		0.23	0.07
Mother marital status: single, living with partner	0.263	251	0.212	184		-0.17	0.24
Mother marital status: married	0.215	251	0.196	184		-0.07	0.75
Mother marital status: divorced/separated	0.052	251	0.027	184		-0.41	0.25
Mother marital status: other or unknown	0.044	251	0.043	184		-0.02	0.92
Mother health is good or better	0.884	251	0.946	184		0.50	0.02
Mother depression (CESD)	0.7 (0.4)	251	0.7 (0.4)	184	-0.07		0.33
Cigarettes per week during pregnancy	5.4 (22.5)	249	2.9 (11.4)	183	-0.13		0.11
Alcohol drinks per week during pregnancy	0.1 (0.6)	249	0.1 (0.6)	184	-0.01		0.97
Number of children born to mother	2.4 (1.3)	251	2.5 (1.4)	184	0.11		0.29
Number of adults in household	2.2 (1.1)	251	2.0 (0.9)	184	-0.19		0.05
Biological father lives in household	0.378	251	0.288	184		-0.25	0.05
Household combined income	$22,739 (20,875)	238	$20,213 (14,402)	168	-0.14		0.15
Household income unknown	0.052	251	0.087	184		0.33	0.17

Household net worth	-$904 (41,220)	222	-$2,296 (13,761)	164	-0.04		0.62
Household net worth unknown	0.116	251	0.109	184		-0.04	0.85

Joint Test: Chi^2 (26)= 36.10, p-value= 0.09, n=435 (includes all observations. Standard joint test estimate drops 6 observations due to collinearity in a small number of observations with values for child's weight unknown, gestational age unknown, and mother's alcohol use unknown.)

Notes: "sd" = standard deviation. P-values were derived from a series of ordinary least squares bivariate regressions in which each respective baseline characteristic was regressed on the treatment status indicator using robust standard errors and site-level fixed effects. The bivariate regressions were also run without site-level fixed effects, and the p-values differed on average by 0.021 and result in no difference in substantive understanding or statistical significance interpretation. The p-values without fixed effects do not appear in the table. The joint test of orthogonality was conducted using a probit model with robust standard errors and site-level fixed effects. Standardized mean differences were calculated using Hedge's g for continuous variables and Cox's Index for dichotomous variables. If there were more than 10 missing cases for a covariate, missing data dummies were included in the table and the joint test. If there were fewer than 10 cases missing, missing data dummies were not included in the table but were included in the joint test; additionally, the joint test imputes mean values for missing variables. Chi-square tests of independence were conducted for the two categorical variables: mother race/ethnicity and mother marital status. For both tests, p>0.05. All respondents with missing data on gestational age, child's weight, and maternal alcohol use are in the low-cash group. We present the results of the joint test that include these observations and exclude these variables for the full sample (which would be dropped in the standard test due to collinearity). If we instead remove these observations from the sample for the joint-test, the sample for the joint-test is slightly reduced, and the estimates are as follows: Joint Test: $Chi^2(26) = 35.62$, p-value = 0.10, n = 429.

SI2. EEG data processing and analysis

EEG was analyzed using the EEGLAB toolbox (2), MATLAB (The MathWorks, Natick, MA), and a low-density version of the MADE pipeline (3) known as the miniMADE pipeline (4). Data were high-pass filtered at 0.3 Hz and low-pass filtered at 50 Hz. Then, data were segmented into epochs of 1 s with 50% overlap between epochs. Epochs were baseline corrected to the mean voltage of each epoch. To remove ocular artifact, a voltage threshold rejection (+/- 250 μV) was applied to two frontal channels (FP1, FP2). If both frontal electrodes exceeded the voltage threshold of +/- 250 μV in an epoch, that epoch was removed from processing. For the remaining channels, those channels containing artifact in each epoch were identified using three criteria: a voltage threshold (+/- 250 μV), a flat channel threshold (range < 1 microvolt for at least half of the epoch), and a jump channel threshold (increases greater than 50 microvolts from sample to sample). Finally, data were re-referenced to an average of T7 and T8.

Following preprocessing, thresholds were applied to ensure adequate artifact-free data remained for each participant prior to power decomposition. First, consistent with previous studies (5), at least 80% (16 out of 20) of electrodes were required to contribute usable data for any given epoch. Second, split-half reliabilities were computed and examined and a cutoff of 20 epochs was

selected so that each band had at least good (>.8) split-half reliability (for more information see Troller-Renfree et al., 2021). Epochs with fewer than 16 artifact-free electrodes and participants with fewer than 20 artifact-free epochs were excluded from further analysis (see CONSORT Diagram in SI1 for more information on participant exclusion). After data cleaning was completed, the mean number of epochs per participant was 286.5 (for the low-cash gift group: M = 288.2, SD = 183.7; for the high-cash gift group: M = 284.3, SD = 189.2).

A Fast Fourier Transformation (FFT) with a 1-second Hanning window was applied to the epoched data (See SI9 to see results when power spectra are log-transformed). Consistent with other infant studies (5, 6), absolute spectral power (μV2) was computed for the theta (3-5 Hz), alpha (6-9 Hz), beta (13-19 Hz), and gamma frequency ranges (21-45 Hz) (to see group differences [z-scores] by single-Hz bins, see Figure 1). Additionally, relative power was computed by dividing the absolute power within one frequency band (e.g., theta) by total absolute power from all frequency bands (theta, alpha, beta, and gamma). Analysis code is available at https://github.com/ChildDevLab.

SI3. Differences between absolute vs. relative power

As discussed in the main text, “absolute power” refers to the brain activity measured across the scalp. Absolute power is typically measured across the frequency spectrum, either in different individual frequency bins, or averaged across individual frequencies within a certain frequency band. “Relative power” refers to the proportion of voltage in one frequency band as it relates to the total power across all bands.

As expected based on the 1/f shape of the EEG power spectrum, the present study reported that absolute theta band power was larger in magnitude than absolute power in the alpha, beta, and gamma bands (see Table 2). That is, theta power makes up a greater proportion of total power than do any of the other bands. Due to this difference in magnitude between the lower and higher frequency bands -- and because relative power is a proportion of power in one band to total power – relative power can be more sensitive to differences in lower bands versus upper bands (6, 7). In our case, we observed no major differences in absolute theta power

between the two groups. In addition, because absolute values of the low-frequency and high-frequency bands are positively correlated, we might expect that standardized group differences in relative power in each mid-to-high-frequency band would be smaller in magnitude, as reported in the main text.

We also note that, in the literature linking SES to brain activity, income has been linked to absolute power (6–12) more commonly than to relative power (10–13). While some studies show associations between income and absolute, but not relative power (6, 7), we are not aware of any studies showing the reverse pattern. Likewise, the literature linking brain activity to language, cognitive, and behavioral outcomes has also more commonly examined absolute power (9, 14–18). However, some studies have found links between these outcomes and both absolute and relative power, or relative power alone (19–21). It is not uncommon for the magnitude of results to differ depending on the type of power examined – particularly when there is a substantial amount of between-subject variation in the magnitude of absolute power values. In our case, our findings generally suggest that the Baby's First Years poverty reduction intervention had a greater impact on mid-to-high-frequency absolute power compared with relative power. However, as the past correlational literature linking income with EEG outcomes is inconsistent in terms of the power type, frequency band, and brain regions examined, further study and replication is needed.

SI4. Preregistration and hypothesis testing

As a randomized control trial, the Baby's First Years project preregistered its analyses with ClinicalTrials.gov (Identifier: NCT03593356) in 2018. At that time, EEG-based analyses were preregistered in three bands: theta, alpha, and gamma, with a multiple comparison correction for both absolute and relative power in these three bands. Given the expectation of at least 80% retention of our initial sample of 1,000 participants, we were powered to detect effect sizes of .21 or greater, and we hypothesized that EEG effects would be within this range. These bands were selected because, at the time of preregistration, the evidence we were aware of from several small-scale correlational studies linked income to differences in resting EEG power in those

bands (6, 8, 13), but not in the beta band. Thus, beta activity was not originally preregistered in 2018, owing to sparse evidence at the time on its association with income. However, between 2018 and the present investigation, evidence has emerged linking income to beta activity, including from the first and senior authors' lab (7, 11).

When we began to analyze data for the current paper, the authors agreed that evidence justified positive group-difference hypotheses related to beta power, and they updated their analytic plan to reflect this. To investigate whether the addition of beta power affected our key results, we compared estimates of group differences in power when multiple testing adjustments did and did not include beta power. Results showed that Westfall-Young adjusted p-values changed minimally (Δp = .00 to .02) and significance (p<.05) did not change (see Table SI4.1 for preregistered Westfall-Young adjusted p-values).

Table SI4.1. Preregistered cash-gift treatment effects on EEG power with various multiple comparison adjustments.

	Effect Size (from Table 2)	Westfall-Young Adjusted p-value (from Table 2; adjustment for alpha, beta, gamma and theta)	Westfall-Young Adjusted p-value (preregistration; adjusted only for alpha, gamma and theta)	N
Absolute Alpha	0.17	0.12	0.12	435
Absolute Gamma	0.23	0.12	0.12	435
Absolute Theta	0.02	0.84	0.84	435
Relative Alpha	0.16	0.31	0.31	435
Relative Gamma	0.16	0.31	0.31	435
Relative Theta	-0.21	0.17	0.15	435

Notes: Effect size (column 1) was computed by dividing the covariate-adjusted treatment effect with the standard deviation of the EEG sample low-cash group. The Westfall-Young adjustment from the main text (column 3), adjusts for the four frequency bands (theta, alpha, beta, gamma) for absolute power into one family and the four frequency bands (theta, alpha, beta, gamma) for relative power were placed into a second family. The Westfall-Young adjustment from the preregistered analyses (column 4), adjusts for the three frequency bands (theta, alpha, gamma) for absolute power into one family and the three frequency bands (theta, alpha, gamma) for relative power were placed into a second family The p-values for both Westfall-Young Adjustments (columns 3 and 4) are associated with the treatment coefficient and effect size in a regression with site-level fixed effects and covariates. Models include the following maternal self-report covariates from the BFY baseline survey conducted at the time of enrollment: mother's age, completed maternal schooling, household income, net worth, general maternal health, maternal mental health, maternal race and ethnicity, marital status, number of adults in the household, number of other children born to the mother, maternal smoking during pregnancy, maternal alcohol consumption during pregnancy, father living with the mother, child's sex, child's birth weight, child's gestational age at birth. Models also control for child's age at interview (in months), and the total number of usable epochs. Missing data for covariates impute the mean value from the EEG analytic sample. Relative power calculatd at the child level.

SI5. Weighted analyses to adjust for selection into the EEG sample

We constructed two types of weights to assess potential differences that may have resulted from selection into the EEG sample, using the Toolkit for Weighting and Analysis of Nonequivalent Groups (TWANG) (22). Broadly, TWANG uses generalized boosted models to flexibly estimate propensity scores and analytic weights.

We first constructed Inverse Probability of Treatment Weights (IPTW), which are intended to estimate the average treatment effect on the treated (ATT). In this approach, participants from the low-cash gift group with usable EEG data are weighted by the odds of being in the high-cash gift group with EEG data, thereby creating a weighted sample in which the low-cash and high-cash gift groups have similar baseline characteristics. We use these weights to assess the sensitivity of our results to any imbalance in baseline characteristics between the low-cash and high-cash gift groups in the EEG sample (Table SI5.1).

Additionally, we created a set of non-response weights intended to adjust for missing EEG data. These weights adjust by the inverse probability of providing enough usable EEG data to be included in our EEG sample; they result in the EEG sample having characteristics similar to the full age-1 BFY analytic sample, including all participants who contributed data, either in-person or over the phone (N=931; see Table SI5.1.).

While the overall pattern of results is robust to both weighting adjustments, the magnitude of our estimates is somewhat sensitive to the adjustments, particularly the IPTW-ATT weights. Specifically, when applying the IPTW weights, designed to address an imbalance between high-cash and low-cash gift groups, the magnitude of estimates decreases for most power bands, suggesting our estimates may be sensitive to some of the observed imbalance between high-cash and low-cash gift groups. When applying the non-response weights, our results are broadly similar, with a slight decrease in the magnitude of effect sizes. These weights are intended to assess whether the same pattern of results might hold had the EEG sample had characteristics similar to the full age 1 BFY analytic sample. Though this suggests we might expect broadly

similar results, we cannot know for certain whether the results presented here would have generalized to the full sample.

Table SI5.1 Cash-gift treatment effect size estimates for base and covariate-adjusted models, applying no weights, inverse probability of treatment weights – average treatment effect on the treated (IPTW-ATT), and non-response weights – average treatment effect (NRW-ATE).

	Unweighted (Taken from Table 2)				IPTW-ATT		NRW-ATE		
	Low-Cash EEG Sample mean	High-Cash EEG Sample mean	Effect Size (base)	Effect Size (with covariates)	Effect Size (base)	Effect Size (with covariates)	Effect Size (base)	Effect Size (with covariates)	Unweighted N
Absolute Alpha	7.441	7.667	0.07	0.17	0.07	0.13	0.07	0.17	435
Absolute Beta	1.874	2.167	0.19	0.26	0.18	0.17	0.13	0.21	435
Absolute Gamma	0.986	1.137	0.16	0.23	0.11	0.13	0.14	0.21	435
Absolute Theta	40.268	38.887	-0.04	0.02	-0.05	-0.01	-0.02	0.04	435
Relative Alpha	0.148	0.152	0.09	0.16	0.13	0.18	0.06	0.13	435
Relative Beta	0.038	0.042	0.15	0.19	0.13	0.12	0.07	0.12	435
Relative Gamma	0.020	0.022	0.11	0.16	0.07	0.09	0.07	0.12	435
Relative Theta	0.794	0.784	-0.14	-0.21	-0.14	-0.17	-0.08	-0.15	435

Notes: This table shows the presented ITT effects (columns 5-8) weighted to adjust for possible biases in the EEG subsample. The unweighted columns (3-4) show ITT effect size estimates for base and covariate-adjusted models, applying no weights. IPTW-ATT signifies Inverse Probability of Treatment Weights (IPTW), which are intended to estimate the average treatment effect on the treated (ATT). NRW-ATE signifies inverse probability weights intended to adjust for missing EEG data. These weights estimate the Average Treatment Effect (ATE) across the full Age 1 analytic sample. The IPTW-ATT present a weighted sample in which the low- and high-cash gift groups in the EEG sample have similar baseline characteristics (columns 6-7). The NWR-ATE weights adjust so that the EEG sample has characteristics similar to the full age 1 BFY analytic sample, including all participants who contributed data, either in-person or over the phone (N=931). Base and covariate-adjusted effect sizes are presented for the three different models. Effect sizes for base models are computed by dividing the treatment effect for a model including only a treatment indicator and site-level fixed effects by the standard deviation of the low-cash EEG sample. Effect size for covariate-adjusted models are computed by dividing the treatment effect for a model including site-level fixed effects and covariates by the standard deviation of the low-cash EEG sample. Weighting models use baseline covariates to estimate propensity scores and create analytic weights. Balance diagnostics suggest weighting was successful at reducing observed baseline imbalance in measured characteristics.

SI6. Effect of the cash-gift treatment on EEG power by region

The preregistered findings presented in the main manuscript detailed how a monthly unconditional cash gift changed whole-brain activity in four bands (theta, alpha, beta, and gamma). While whole-brain effects are informative, they are not most commonly examined in the EEG literature. Previous EEG studies examining the association between socioeconomic status (SES) and brain activity have all examined how EEG power differed not only by power band, but also by the brain region over which electrodes were placed (e.g., frontal, central, parietal, etc.).

Past correlational research has reported socioeconomic disparities in regional brain activity in the theta (8–10), alpha (8–10, 12), beta (7, 11, 12), and gamma bands (6, 11). Within the theta band, higher SES has been related to less theta power in the frontal, temporal, and parietal regions (8–10). For the alpha band, higher SES has been related to more alpha power in all regions, including frontal (8–10, 12), temporal (8, 10), central (9, 10, 12), parietal (10), and occipital (8, 10, 12) regions. Likewise, for beta power, higher SES has been related to more power in the frontal (11), temporal, (7, 12) central (7, 11, 12), parietal (11), and occipital (7, 12) regions. Finally, in the gamma band, higher SES has been related to more power in the frontal (6, 11), central (11), and parietal (11) regions. Broadly, the majority of these studies suggest that greater socioeconomic resources are related to less low-frequency power (theta) and more mid-to-high-frequency power (alpha, beta, gamma), but also suggest that the observation of these effects may vary by brain region. As a result, considering regional differences may add important, novel information to the whole-brain findings reported in the main text.

To investigate regional effects, we averaged available data from electrodes in each of four regions: frontal, central, parietal, and occipital (see Figure SI6.1). Of note, the temporal electrodes served as our reference electrodes, so it is impossible to investigate group differences in these regions. For statistical analyses, Westfall-Young corrections were applied within each band (correcting for the four regions as a family).

Table SI6.1 shows ITT estimates by region within each band, before and after adjustments for baseline covariates and multiple comparisons. First, for alpha power, the high-

cash gift group showed more absolute power than the low-cash gift group in the frontal region (effect size = 0.19, *ß* = 0.804, *p* = 0.05); however, this effect did not survive Westfall-Young adjustment (p =.12). No significant regional differences in relative alpha power were present.

For beta power, the high-cash gift group showed more absolute power in the frontal (effect size = 0.32, *ß* = 0.460, *p* = 0.01) and central (effect size = 0.28, *ß* = 0.585, *p* = 0.02) regions, and more relative power in the frontal region (effect size = 0.24, *ß* = 0.007, *p* = 0.04), as compared with the low-cash gift group. Differences in frontal (*p* = 0.02) and central (*p* = 0.05) absolute power remained significant after Westfall-Young adjustment, while differences in relative power fell to the margins of significance (*p* =0.10).

For gamma power, group differences in absolute power were observed in the frontal (effect size = 0.26, *ß* = 0.238, *p* = 0.02) and central regions (effect size = 0.26, *ß* = 0.317, *p* = 0.04). The frontal effect remained significant (frontal: *p* =.04), whereas the central effect fell to the margins of significance (central: *p* = .08) after Westfall-Young adjustment for multiple comparisons. No significant regional differences were observed in relative gamma power.

Finally, for theta power, there were no regional effects for absolute power. For relative power, the high-cash gift group showed less theta activity in the frontal region (effect size = -0.25, *ß* = -0.018, *p* = 0.04); however, after Westfall-Young adjustment, this difference fell to the margins of significance (*p* = 0.09).

Altogether, our regional analyses show patterns consistent with the whole-brain analyses reported in the main text, with the high-cash gift group showing more absolute mid-to-high-frequency power, as well as some evidence for less low-frequency relative power, as compared with the low-cash gift group. However, importantly, regional analyses provide further evidence that these whole-brain differences are driven by larger, statistically significant differences in the frontal and central areas of the brain. This pattern is consistent with a number of previous papers that have shown that increased socioeconomic resources are related to increased absolute mid-to-high-frequency power in the frontal and central regions (6, 8–10, 12) as well as decreased

relative low-frequency power in frontal regions (10). The frontal region is of particular interest given that increased mid-to-high-frequency power in the frontal region has been related to subsequent higher language (14, 15), cognitive (16, 17, 23), and socioemotional (18) scores.

Figure SI6.1. Electrodes by region.

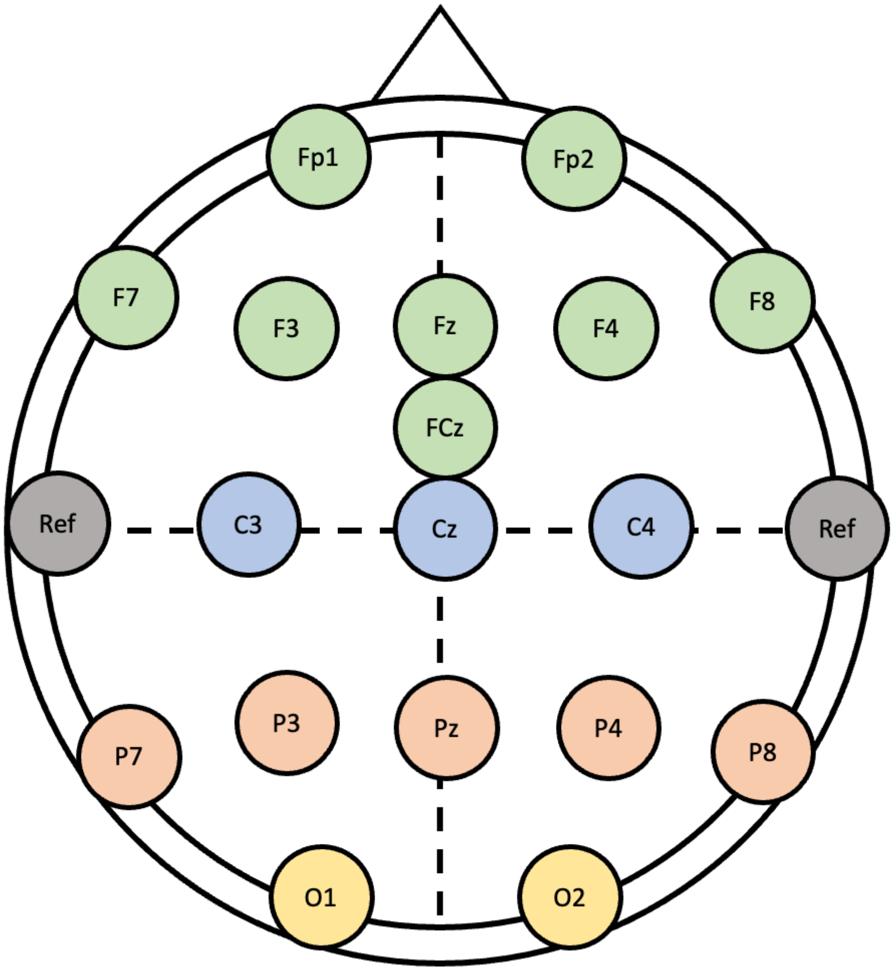

Electrode groupings by region. Electrode locations are approximate and follow the conventional EEG electrode locations. Each region is designated both by color and leading letter of electrode (e.g., P or F). Frontal is shown in green, central is shown in blue, parietal is shown in orange, and occipital is shown in yellow.

Table SI6.1. Cash-gift treatment effects on EEG power by region.

	Low-Cash Gift Group mean	High-Cash Gift Group mean	OLS w/FE	OLS w/FE w/covariates	Effect Size	p-value	Westfall-Young Adjusted p-value	N
				**Absolute Power**				
				Alpha				
Central	6.897	7.190	0.354	0.694	0.14	0.14	0.28	435
Frontal	7.085	7.456	0.436	0.804	0.19	0.05	0.12	435
Occipital	10.352	10.477	0.187	0.941	0.14	0.17	0.28	435
Parietal	7.172	7.166	0.074	0.513	0.11	0.20	0.28	435
				Beta				
Central	1.998	2.431	0.443	0.585	0.28	0.02	0.05	435
Frontal	1.762	2.131	0.384	0.460	0.32	0.01	0.02	435
Occipital	1.901	1.940	0.053	0.202	0.11	0.22	0.22	435
Parietal	1.966	2.158	0.206	0.324	0.17	0.09	0.13	435
				Gamma				
Central	1.043	1.264	0.223	0.317	0.26	0.04	0.08	435
Frontal	0.968	1.146	0.183	0.238	0.26	0.02	0.04	435
Occipital	0.887	0.922	0.038	0.108	0.11	0.31	0.31	435
Parietal	1.020	1.130	0.114	0.181	0.17	0.14	0.20	435
				Theta				
Central	36.067	35.329	-0.307	1.077	0.04	0.64	0.92	435
Frontal	35.860	35.338	-0.122	1.069	0.05	0.56	0.92	435
Occipital	58.813	55.890	-2.607	-0.646	-0.01	0.86	0.93	435
Parietal	42.424	39.898	-2.037	-0.674	-0.02	0.77	0.93	435
				**Relative Power**				
				Alpha				
Central	0.150	0.151	0.001	0.003	0.06	0.61	0.62	435
Frontal	0.156	0.162	0.005	0.008	0.18	0.14	0.30	435
Occipital	0.148	0.150	0.002	0.006	0.13	0.29	0.43	435
Parietal	0.138	0.142	0.004	0.006	0.16	0.17	0.31	435
				Beta				
Central	0.044	0.049	0.005	0.006	0.18	0.13	0.25	435
Frontal	0.040	0.046	0.006	0.007	0.24	0.04	0.10	435
Occipital	0.029	0.030	0.001	0.002	0.08	0.40	0.39	435
Parietal	0.038	0.042	0.003	0.004	0.14	0.20	0.29	435
				Gamma				
Central	0.023	0.025	0.002	0.003	0.13	0.28	0.45	435

Frontal	0.022	0.025	0.003	0.003	0.18	0.11	0.22	435
Occipital	0.014	0.014	0.000	0.001	0.06	0.55	0.54	435
Parietal	0.020	0.022	0.001	0.002	0.10	0.38	0.50	435
				Theta				
Central	0.783	0.775	-0.007	-0.012	-0.14	0.24	0.35	435
Frontal	0.782	0.768	-0.014	-0.018	-0.25	0.04	0.09	435
Occipital	0.809	0.806	-0.003	-0.009	-0.12	0.26	0.35	435
Parietal	0.803	0.795	-0.008	-0.013	-0.17	0.14	0.25	435

Notes: "OLS" = Ordinary Least Squares; Effect Size was computed by dividing the covariate-adjusted treatment effect (column 4) with the standard deviation of the EEG sample low-cash group. Unadjusted p-values (column 6) and Westfall-Young adjusted p-values (column 7), which adjust for multiple hypothesis testing, are both reported. For the Westfall-Young adjustment, each absolute and relative frequency band was put into its own family; therefore, this adjusts within a specific band (e.g., Absolute Alpha). Both sets of p-values are associated with the treatment coefficient and effect size in a regression with site-level fixed effects and covariates. Models include the following pre-registered maternal self-report covariates from the BFY baseline survey conducted at the time of enrollment: mother's age, completed maternal schooling, household income, net worth, general maternal health, maternal mental health, maternal race and ethnicity, marital status, number of adults in the household, number of other children born to the mother, maternal smoking during pregnancy, maternal alcohol consumption during pregnancy, father living with the mother, child's sex, child's birth weight, child's gestational age at birth. Models also control for child's age at interview (in months), and the total number of usable epochs. Missing data for covariates impute the mean value from the EEG analytic sample. Absolute power within each band is calculated by averaging the power band measure from electrodes within each region (i.e., Occipital Absolute Alpha averages measures of Absolute Alpha from the two electrodes in the Occipital region). Relative power is calculated as a ratio of an absolute measure and the total power within each region.

SI7. Cash-gift Treatment Impacts on a Post-Hoc Composite Index of Mid-to-High-Frequency Brain Activity

As a robustness check of the effects of the cash-gift intervention on infant brain activity, we constructed a single post-hoc composite measure that aggregated across the portion of the spectrum defined by the three mid-to-high-frequency bands. Because this approach is focused on estimating intent-to-treat differences in a single index score, there is no need for multiple-testing adjustments. To construct this index of mid-to-high-frequency power, we summed the absolute power values across the entire mid-to-high-frequency portion of the power spectrum, in each single-Hz bin from 6 Hz – 49 Hz. In this way, we can assess the intent-to-treat impact of the cash gifts on a single measure of absolute power, employing data from across the mid-to-high-frequency (including alpha, beta, and gamma) regions of the power spectrum. We acknowledge that this approach ignores functional definitions of these mid-to-high-frequency bands, the overall

shape of the power spectrum, as well as the data shape within each of the individual frequency bands. Rather, we use this summary index approach (24, 25) as a useful complement to addressing problems encountered with statistical power when each band is considered separately.

ITT analyses of the mid-to-high-frequency power index in Table SI7.1 show that the high-cash gift group had greater composite mid-to-high-frequency absolute power than the low-cash gift group (effect size = 0.25, ß = 13.35, p = 0.02).

Table SI7.1 Cash-gift treatment effects on summed high frequency single-hz bins (6-49)

	Low-Cash Gift Group mean (sd)	High-Cash Gift Group mean (sd)	OLS with site fixed effects (se)	OLS with site fixed effects and covariates (se)	Effect Size	p-value	N
Sum of High Frequency Single-Hz Bins (6-49)	79.018 (52.518)	86.888 (64.442)	8.414 (5.591)	13.354 (5.632)	0.25	0.02	435

Notes: "OLS" = Ordinary Least Squares; "sd" = standard deviation; "se" = standard error. The sum of high frequency bins sums single-Hz bins beginning with those in the alpha band. It includes each bin, regardless of whether the bin is included in one of the power bands. OLS = Ordinary Least Squares. Effect size (column 5) was computed by dividing the covariate-adjusted treatment effect (column 4) with the standard deviation of the EEG sample low-cash group. Unadjusted p-values (column 6) are reported. Models include the following maternal self-report covariates from the BFY baseline survey conducted at the time of enrollment: mother's age, completed maternal schooling, household income, net worth, general maternal health, maternal mental health, maternal race and ethnicity, marital status, number of adults in the household, number of other children born to the mother, maternal smoking during pregnancy, maternal alcohol consumption during pregnancy, father living with the mother, child's sex, child's birth weight, child's gestational age at birth. Models also control for child's age at interview (in months), and the total number of usable epochs. Missing data for covariates impute the mean value from the EEG analytic sample. All models are estimated using robust standard errors. Relative power is calculated at the child level. Standard errors in parentheses for OLS models (columns 4 and 5). Standard deviations are shown in parentheses for group means.

SI8. Cash-gift treatment impacts on maternal report of infant language milestones in the EEG sample and the full analytic sample

As a behavioral complement to the EEG-based measures of brain activity, we conducted ITT analyses of mothers' reports of their infants' achievement of age-appropriate language milestones. We hypothesized that mothers randomized to the high-cash gift group would report that their infants were meeting more age-appropriate language milestones compared with infants of mothers randomized to the low-cash gift group.

Infant language milestones were assessed using the Ages and Stages Questionnaire (ASQ-3) Communication subscale (26). The ASQ-3 uses maternal report to screen for developmental delays. The Communication subscale of the ASQ-3 includes six items measuring children's developmentally-appropriate language skills (e.g., "Does your baby make two similar sounds, such as "ba-ba," "da-da," or "ga-ga"?"). The items in the ASQ-3 differ by child age, and mothers were administered the correct form based on the child's age at the time of the interview. For each item, mothers reported whether their infant demonstrated a given skill regularly, sometimes, or not yet. Scores were calculated by summing the item scores. Raw scores were then z-scored using age-normed means and standard deviations for the ASQ-3. Higher z-scores indicated that the child demonstrated higher levels of developmentally-appropriate language skills relative to the skills of same-aged peers. The ASQ-3 has shown strong concurrent validity with the Battelle Developmental Inventory screener (86% precent agreement), two-week test-retest reliability (r =.75-.82), inter-observer reliability (r =.43-.69), and internal consistency (α =.51 to .87)(26).

For the n=435 subset of mother/infant dyads who contributed usable EEG data, ITT analyses revealed that infants in the high-cash gift group had achieved more age-appropriate language milestones than infants in the low-cash gift group (ß = 0.189, p = 0.03, effect size = 0.22). However, results for the full sample (n=900), showed smaller and, despite the larger

sample size, nonsignificant differences (*ß* = 0.075, *p* = 0.21, effect size = 0.08) (see SI10 for associations between EEG power and infant language milestones).

These differences may stem from several sources. First, moving the collection of the ASQ-3 from in-person to over the phone may have elicited different responses from mothers, obscuring our ability to detect group differences. However, mean language milestone scores did not differ between in-person and phone-based collection (*p* = 0.67), rendering this possibility less likely.

Second, impacts of the cash gift on children's language milestone development may have differed in meaningful ways before the pandemic compared with after the onset of the pandemic. For example, an examination of the raw means reveals that the high-cash gift group mean score was similar before the pandemic (M = .299) compared with after the onset of the pandemic (M = .271), whereas the low-cash gift mean was somewhat lower before the pandemic (M = .168) compared with after pandemic onset (M = .239). Such a pattern could potentially be explained if the high-cash gift group experienced greater language input than the low-cash gift group before the pandemic, but language input was equalized after the onset of the pandemic when all children, regardless of group, may have been more likely to spend time at home with family members.

Finally, because these participants from whom usable EEG data were obtained comprised a non-random subset of the larger BFY sample, it may be that children who successfully completed EEG data collection differ meaningfully from those who did not, in a way that interacted with the cash gift to predict language milestones. This possibility is rendered somewhat less likely from the NRW-ATE analyses described in SI5, which suggested that the findings in the EEG subgroup would have been similar had the EEG sample had characteristics similar to the full age 1 BFY analytic sample.

This uncertainty about why treatment group differences in the ASQ were evident in the EEG subgroup but not in the full sample suggests that caution in interpreting the main findings of this manuscript is merited. In particular, it suggests the possibility that some of the brain activity

findings may not have generalized to the portion of the sample surveyed following the onset of the pandemic. In future waves of the study, which are intended to take place once it is safe and feasible to assess all participants in-person, brain activity will be assessed among the full sample. Notably, the measure of children's language milestones was limited to maternal report; subsequent waves of data collection will directly measure children's cognitive and behavioral outcomes in a laboratory setting, providing a much more sensitive measure of child development that is free from the bias of maternal reporting.

SI9. Logged vs unlogged power spectra

There is some debate within the field of psychophysiology as to whether power spectra should be log transformed prior to band power computation. For primary analyses, participants' power spectra were not log-transformed, because we lacked a theoretical reason suggesting that a log-transformation would impact ITT estimates. However, as a robustness check, we also computed and examined log-transformed band power. Group differences were qualitatively similar to those presented in Table 2, although effects sizes were somewhat smaller, and results fell below conventional levels of statistical significance (see Table SI9.1 for regression tables).

Table SI9.1. Regressions estimating cash-gift treatment effects on log-transformed EEG power.

	Low-cash Gift Group mean (sd)	High-cash Gift Group mean (sd)	Ordinary least squares with fixed effects (se)	Ordinary least squares with fixed effects and covariates (se)	Effect Size	p-value	Westfall-Young adjusted p-value	N
Absolute Alpha (log)	0.808 (0.223)	0.824 (0.208)	0.020 (0.020)	0.043 (0.022)	0.19	0.05	0.14	435
Absolute Beta (log)	0.382 (0.194)	0.403 (0.215)	0.023 (0.019)	0.037 (0.020)	0.19	0.06	0.14	435
Absolute Gamma (log)	0.236 (0.157)	0.252 (0.175)	0.016 (0.016)	0.026 (0.017)	0.16	0.13	0.23	435
Absolute Theta (log)	1.453 (0.256)	1.463 (0.198)	0.015 (0.021)	0.034 (0.023)	0.13	0.15	0.23	435

Relative Alpha (log)	0.279 (0.028)	0.279 (0.029)	0.001 (0.003)	0.003 (0.003)	0.11	0.31	0.49	435
Relative Beta (log)	0.125 (0.042)	0.129 (0.045)	0.004 (0.004)	0.007 (0.004)	0.16	0.13	0.29	435
Relative Gamma (log)	0.075 (0.039)	0.078 (0.041)	0.003 (0.004)	0.004 (0.004)	0.11	0.28	0.49	435
Relative theta (log)	0.521 (0.080)	0.514 (0.083)	-0.008 (0.008)	-0.014 (0.009)	-0.18	0.10	0.24	435

Notes: "OLS" = Ordinary Least Squares; "sd" = standard deviations; "se" = standard errors. In contrast to the results presented in Table 2, the power spectrum measures in this table were log-transformed. Effect size (column 5) is computed by dividing the covariate-adjusted treatment effect (column4) with the standard deviation of the EEG sample low-cash group. Unadjusted p-values (column 6) and Westfall-Young adjusted p-values (column 7), which adjust for multiple hypothesis testing, are both reported. For the Westfall-Young adjustment, the four frequency bands (theta, alpha, beta, gamma) for absolute power are placed into one family and the four frequency bands (theta, alpha, beta, gamma) for relative power were placed into a second family. These p-values are associated to the treatment coefficient and effect size in a regression with site-level fixed effects and covariates. Models include the following maternal self-report covariates from the BFY baseline survey conducted at the time of enrollment: mother's age, completed maternal schooling, household income, net worth, general maternal health, maternal mental health, maternal race and ethnicity, marital status, number of adults in the household, number of other children born to the mother, maternal smoking during pregnancy, maternal alcohol consumption during pregnancy, father living with the mother, child's sex, child's birth weight, child's gestational age at birth. Models also control for child's age at interview (in months), and the total number of usable epochs. Missing data for covariates impute the mean value from the EEG analytic sample. Relative power is calculated at the child level. Robust standard errors are given in parentheses.

SI10. Associations between EEG power and language milestones

A growing body of research suggests that resting EEG power is associated with language skill (7, 14–16). We investigated whether this was in the case in the EEG sample by estimating correlations between EEG power and language milestone scores (see Table SI10.1). Results showed no significant statistical associations between language milestones and either absolute or relative power in any whole-brain or regional analysis (p's > .05).

The nonsignificant association between EEG power and language milestones may stem from several sources. First, our language milestone assessment, the ASQ-3 communication scale, relies on maternal report of six items. While this measure is valid and reliable as a broad screener for delayed language milestones, it does not capture the fine-grained variability in child language development that is likely necessary to see brain-behavior associations. Furthermore, parental report on infant language development can be flawed and influenced by factors other than a child's language skill. For example, parental educational attainment may lead to different response on a language screener (27). Another possibility is that the high-cash gift group may have engaged more frequently with their children than the low-cash gift group, providing a greater

opportunity for the high-cash gift group to observe language milestones in their children. Finally, relations between EEG power and language skill tend to get stronger across development (7, 14–16). Indeed, it is not uncommon that brain activity in infancy fails to predict concurrent language skill, but instead predicts future language ability (16). Thus, while we did not detect a significant correlation between whole-brain EEG power and language milestones, it is possible that such an association would exist with more sensitive measures, and/or will emerge at later waves of data collection.

Table SI10.1. Associations between EEG power and language milestones

	Correlation (*r*) with Language Milestones (Standardized)	p-value	N
Absolute Alpha	0.041	0.39	431
Absolute Beta	-0.007	0.88	431
Absolute Gamma	-0.014	0.77	431
Absolute Theta	0.028	0.56	431
Relative Alpha	0.041	0.39	431
Relative Beta	-0.029	0.55	431
Relative Gamma	-0.030	0.54	431
Relative Theta	-0.003	0.95	431
Index of High-Frequency Power	0.005	0.92	431

Notes: This table shows the correlation between whole-brain EEG power and language milestones. Pearson's correlation coefficients are reported in Column 2 and associated p-values are reported in Column 3. The difference in sample size is due to missing ASQ measures for four children in the EEG sample.

References

1. K. G. Noble, *et al.*, Baby's First Years: Design of a Randomized Controlled Trial of Poverty Reduction in the U.S. *Pediatrics* (2021).

2. A. Delorme, S. Makeig, EEGLAB: an open source toolbox for analysis of single-trial EEG dynamics including independent component analysis. *J. Neurosci. Methods* **134**, 9–21 (2004).

3. R. Debnath, *et al.*, The Maryland Analysis of Developmental EEG (MADE) Pipeline. *Psychophysiology* (2020) https:/doi.org/10.1111/psyp.13580.

4. S. V. Troller-Renfree, *et al.*, Feasibility of Assessing Brain Activity using Mobile, In-home Collection of Electroencephalography: Methods and Analysis. *Dev. Psychobiol.* (2021).

5. S. V Troller-Renfree, *et al.*, Infants of Mothers with Higher Physiological Stress Show Alterations in Brain Function. *Dev. Sci.* (2020) https:/doi.org/10.1111/desc.12976 (April 28, 2020).

6. P. Tomalski, *et al.*, Socioeconomic status and functional brain development - associations in early infancy. *Dev. Sci.* **16**, 676–687 (2013).

7. N. H. Brito, *et al.*, Associations among the home language environment and neural activity during infancy. *Dev. Cogn. Neurosci.* **43**, 100780 (2020).

8. G. A. Otero, Poverty, cultural disadvantage and brain development: a study of pre-school children in Mexico. *Electroencephalogr. Clin. Neurophysiol.* **102**, 512–516 (1997).

9. M. J. Maguire, J. M. Schneider, Socioeconomic status related differences in resting state EEG activity correspond to differences in vocabulary and working memory in grade school. *Brain Cogn.* **137** (2019).

10. G. A. Otero, F. . Pliego-Rivero, T. Fernández, J. Ricardo, EEG development in children

with sociocultural disadvantages: a follow-up study. *Clin. Neurophysiol.* **114**, 1918–1925 (2003).

11. S. K. G. Jensen, *et al.*, Associations of socioeconomic and other environmental factors with early brain development in Bangladeshi infants and children. *Dev. Cogn. Neurosci.* **50**, 100981 (2021).

12. G. A. Otero, Eeg spectral analysis in children with sociocultural handicaps. *Int. J. Neurosci.* **79**, 213–220 (1994).

13. T. Harmony, *et al.*, EEG Maturation on Children with Different Economic and Psychosocial Characteristics. *Int. J. Neurosci.* **41**, 103–113 (1988).

14. A. A. Benasich, Z. Gou, N. Choudhury, K. D. Harris, Early cognitive and language skills are linked to resting frontal gamma power across the first 3 years. *Behav. Brain Res.* **195**, 215–222 (2008).

15. Z. Gou, N. Choudhury, A. A. Benasich, Resting frontal gamma power at 16, 24 and 36 months predicts individual differences in language and cognition at 4 and 5 years. *Behav. Brain Res.* **220**, 263–270 (2011).

16. N. H. Brito, W. P. Fifer, M. M. Myers, A. J. Elliott, K. G. Noble, Associations among family socioeconomic status, EEG power at birth, and cognitive skills during infancy. *Dev. Cogn. Neurosci.* **19**, 144–151 (2016).

17. I. A. Williams, *et al.*, Fetal cerebrovascular resistance and neonatal EEG predict 18-month neurodevelopmental outcome in infants with congenital heart disease. *Ultrasound Obstet. Gynecol.* **40**, 304–309 (2012).

18. N. H. Brito, *et al.*, Neonatal EEG linked to individual differences in socioemotional outcomes and autism risk in toddlers. *Dev. Psychobiol.* **61**, 1110–1119 (2019).

19. T. Harmony, *et al.*, Correlation Between EEG Spectral Parameters and an Educational

Evaluation. *Int. J. Neurosci.* **54**, 147–155 (1990).

20. K. A. McLaughlin, *et al.*, Delayed maturation in brain electrical activity partially explains the association between early environmental deprivation and symptoms of attention-deficit/hyperactivity disorder. *Biol. Psychiatry* **68**, 329–36 (2010).

21. R. J. Barry, A. R. Clarke, S. J. Johnstone, A review of electrophysiology in attention-deficit/hyperactivity disorder: I. Qualitative and quantitative electroencephalography. *Clin. Neurophysiol.* **114**, 171–183 (2003).

22. G. Ridgeway, D. McCaffrey, A. Morral, L. Burgette, B. Griffin, “Toolkit for Weighting and Analysis of Nonequivalent Groups: A tutorial for the twang package” (2017).

23. W. C. Corning, R. A. Steffy, E. Anderson, P. Bowers, EEG ?maturational lag? profiles: Follow-up analyses. *J. Abnorm. Child Psychol.* **14**, 235–249 (1986).

24. H. Hoynes, D. W. Schanzenbach, D. Almond, Long-Run Impacts of Childhood Access to the Safety Net. *Am. Econ. Rev.* **106**, 903–34 (2016).

25. J. R. Kling, J. B. Liebman, L. F. Katz, Experimental Analysis of Neighborhood Effects. *Econometrica* **75**, 83–119 (2007).

26. J. Squires, *et al.*, “Ages & Stages Questionnaires ® A Parent-Completed Child Monitoring System THIRD EDITION ASQ-3™” (2009).

27. J. Law, P. Roy, Parental Report of Infant Language Skills: A Review of the Development and Application of the Communicative Development Inventories. *Child Adolesc. Ment. Health* **13**, 198–206 (2008).
